# Extent, regional variation and impact of gynecologist payment models in routine pelvic examinations: a nationwide cross-sectional study

**DOI:** 10.1186/s12905-017-0471-2

**Published:** 2017-11-21

**Authors:** Ingvild Mathiesen Rosenlund, Linda Leivseth, Ingard Nilsen, Olav Helge Førde, Arthur Revhaug

**Affiliations:** 1 Department of Clinical Medicine, UiT The Arctic University of Norway, Tromsø, Norway; 2 Centre for Clinical Documentation and Evaluation, Northern Norway Regional Health Authority, Tromsø, Norway; 3 Department of Obstetrics and Gynaecology, University Hospital of North Norway, Tromsø, Norway; 4 Department of Community Medicine, UiT The Arctic University of Norway, Tromsø, Norway; 5 Division of Surgery, Oncology and Women’s Health, University Hospital of North Norway, Tromsø, Norway

**Keywords:** Routine pelvic examination, Unwarranted examination, Fee-for-service, Regional variation, Ultrasonography, Colposcopy

## Abstract

**Background:**

Based on moderate quality evidence, routine pelvic examination is strongly recommended against in asymptomatic women. The aims of this study was to quantify the extent of routine pelvic examinations within specialized health care in Norway, to assess if the use of these services differs across hospital referral regions and to assess if the use of colposcopy and ultrasound differs with gynecologists’ payment models.

**Methods:**

Nationwide cross-sectional study including all women aged 18 years and older in Norway in the years 2014–16 (2,038,747). Data was extracted from the Norwegian Patient Registry and Statistics Norway. The main outcome measures were 1. The number of appointments per 1000 women with a primary diagnosis of “Encounter for gynecological examination without complaint, suspected or reported diagnosis.” 2. The age-standardized number of these appointments per 1000 women in the 21 different hospital referral regions of Norway. 3. The use of colposcopy and ultrasound in routine pelvic examinations, provided by gynecologists with fixed salaries and gynecologists paid by a fee-for-service model.

**Results:**

Annually 22.2 out of every 1000 women in Norway had a routine pelvic examination, with variation across regions from 6.6 to 43.9 per 1000. Gynecologists with fixed salaries performed colposcopy in 1.6% and ultrasound in 74.5% of appointments. Corresponding numbers for fee-for-service gynecologists were 49.2% and 96.2%, respectively.

**Conclusions:**

Routine pelvic examinations are widely performed in Norway. The variation across regions is extensive. Our results strongly indicate that fee-for-service payments for gynecologists skyrocket the use of colposcopy and increase the use of ultrasound in pelvic examinations of asymptomatic women.

**Electronic supplementary material:**

The online version of this article (10.1186/s12905-017-0471-2) contains supplementary material, which is available to authorized users.

## Background

Based on moderate quality evidence, routine pelvic examination is strongly recommended against in asymptomatic women [1–3], as is screening colposcopy [4] and routine screening for ovarian cancer in asymptomatic women [5–7]. In the Prostate, Lung, Colorectal and Ovarian Cancer Screening Randomized Controlled Trial, including 78,000 women, bimanual examination of the ovaries was discontinued as no ovarian cancer was detected merely by palpation [8]. Use of screening CA 125 and transvaginal ultrasound does not reduce ovarian cancer mortality [8, 9] and is advised against [5, 10]. High rates of colposcopy do not decrease cervical cancer incidence or mortality [11]. False positive screening test results are associated with harm. Women screened for ovarian cancer have a 33% increased risk of oophorectomy [8] and for every screening detected cancer ten women undergo surgery following a false positive ultrasound examination [9]. Ultrasound of the pelvis should not be performed unless clear indications are present [12]. Pelvic examination prior to provision of hormonal contraceptives does not identify women who should avoid these contraceptives and is not recommended as routine practice [13].

The Norwegian public health system is well developed with access for all inhabitants. All citizens have a legal right to equal access to good quality health care [14]. All citizens are entitled to a regular general practitioner who, if necessary, refers the patient to specialized care. Apart from abortions, women themselves cannot make an appointment at a publicly reimbursed gynecologist without a referral.

The municipalities of Norway are allocated into 21 different hospital referral regions. Each region has a defined health enterprise responsible for providing specialized health care for their inhabitants. The health enterprises collaborate with private physicians to varying degrees. In Norway all gynecologists at public hospitals are paid a fixed salary, while a fee-for-service model pays private gynecologists that collaborate with the health enterprises.

## Methods

### Aims

1. To quantify the extent of routine pelvic examinations within specialized health care in Norway.
2. To assess if the use of these services differs across hospital referral regions.
3. To assess if the use of colposcopy and ultrasound differs with gynecologists’ payment models.

### Study design

Nationwide cross-sectional study on routine pelvic examinations within specialized health care in Norway.

### Setting

The Norwegian Patient Registry (NPR) contains health reports on every appointment within publicly funded specialized health care in Norway. Both public hospitals and private gynecologists that collaborate with the health enterprises report diagnoses of every patient appointment to NPR. The appointments are linked to the patients through the personal identification number of all inhabitants of Norway. Data from NPR is used for central planning of specialized health care, activity based financing, quality indicators, and health care research. Through Centre for Clinical Documentation and Evaluation (SKDE) we had access to reports for 2014–16 in addition to annual population statistics from Statistic Norway. One author (LL) had access to indirect personally identifiable data from NPR. All analyses and results are anonymous.

### Participants

We included all Norwegian women aged 18 years and older in Norway in the years 2014–16 (*n* = 2,038,747).

### Variables and data sources

In this study private gynecologists who get public reimbursement are for simplicity called “fee-for-service gynecologists.” The term “fixed salary gynecologists” is used for gynecologists working in public hospitals, as the salaries of these gynecologists are independent of quantity of care. Data from privately out-of-pocket paid gynecologists’ practice is not included in the study as such data is not recorded in Norway.

Both fixed salary and fee-for-service gynecologists used the International Classification of Diseases version 10 (ICD-10) for reports to NPR. The Norwegian versions for 2014–16 were in use during the study years. The three versions are identical for the codes we have studied [15]. All gynecologists also reported the municipality and city district code for the patients’ residency. We used these codes to allocate appointments to the different hospital referral regions.

We defined routine pelvic examination as a primary diagnosis of ICD-10 Z01.4; “Encounter for gynecological examination without complaint, suspected or reported diagnosis.” Cervical screening was defined as a primary diagnosis of the ICD-10 code Z12.4; “Encounter for screening for malignant neoplasm of cervix.”

For hospital appointments colposcopy was defined by the allocation of the code LXE00 (colposcopy) in the 2014–16 versions of The NOMESCO Classification of Surgical Procedures (NCSP) [16]. Ultrasound was defined by the allocation of any of the codes LXDE05 (transvaginal ultrasound), SLXOBK or SLXOAK (transvaginal ultrasound of female pelvic organs) in the 2014–16 versions of the Norwegian Classification of Medical Procedures (NCMP) and the Norwegian Classification of Radiological Procedures (NCRP) [16]. For fee-for-service gynecologists colposcopy and ultrasound were defined either by the same procedure codes or by the allocation of the codes 208 (colposcopy) and 211c (transvaginal ultrasound) in “Tariff for publicly funded private physicians” versions 2014–16 [17]. In addition “complete examination” at a fee-for-service gynecologist was defined by the allocation of the tariff code 4b1 (allowance for complete examination performed by a specialist (after referral)).

Both publicly funded hospitals and fee-for-service gynecologists that get public funding are obligated to report surgical, medical, and radiological procedures according to NCSP, NCMP, and NCRP to NPR. In addition, fee-for-service gynecologists include tariff codes from “Tariff for publicly funded private physicians” in the reports sent to NPR. For each individual tariff code they report they get extra reimbursement. It is known that some fee-for-service physicians underreport NCSP and NCMP codes. Therefore we also included the more thoroughly reported tariff codes to the definition of colposcopy and ultrasound for fee-for-service gynecologists.

### Statistical analysis

We obtained the age-standardized number of appointments for routine pelvic examination per 1000 women in Norway, and for the 21 different hospital referral regions. We also quantified the use of colposcopy and ultrasound in appointments for routine pelvic examination for women examined by gynecologists with fixed salaries and fee-for-service gynecologists, respectively. Differences between provider types were compared with Pearson’s chi-square test. For fee-for-service gynecologists the number of appointments with codes for “Allowance for complete examination performed by a specialist (after referral)” was also examined. All numbers reported are the annual mean for 2014–16, unless otherwise stated.

There were missing data for municipality code in 0.5% (215) of appointments annually. As it is likely that patients do not travel far for routine pelvic examinations, we analyzed these appointments according to the referral region where the examination took place.

In additional analyses, we quantified the extent of cervical screening tests within specialized health care in Norway, and we examined if differences across hospital referral regions in routine pelvic examinations were depended on differences in cervical screening tests. We also examined if the use of colposcopy and ultrasound, and variation across regions in pelvic examinations were depended on registered secondary diagnosis.

We used SAS Enterprise Guide 7.1 [18] to analyze the data.

### Patient involvement

Patients were not formally involved in the planning or conduction of the study. However, the subject under investigation involves all Norwegian adult women including the female authors of this paper.

## Results

The estimated adult female population of Norway in the years 2014–16 was 2,038,747. Nationally there were 22.2 pelvic examinations per 1000 women (Table 1). Of women who received a pelvic examination during the years of investigation, the majority (88.9%) had only one exam. The number of appointments per patient was 1.04 annually and 1.14 during the 3 year period.

**Table 1:** Appointments for routine pelvic examinations in Norway, 2014–16

Measures	2014	2015	2016	Annual mean 2014–16
Patients, n	44,731	41,941	43,644	43,439
Appointments, n	46,619	43,534	45,382	45,178
Appointments per 1000 women	23.1	21.3	22.0	22.2
Age, years, mean/median (range)	47.7/47 (18–98)	47.5/47 (18–101)	47.1/46 (18–100)	47.4/47 (18–101)

**Fig. 1:**
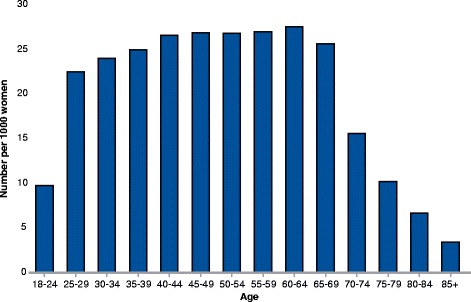
Number per 1000 women receiving routine pelvic examinations by age groups. Annual mean for the years 2014–16

Women aged 25–69 years constituted 87.6% (39,589) of appointments. Annually, 2.6% (38,065) of women aged 25–69 years had a routine pelvic examination, while 0.97% (2231) of younger women and 0.96% (3143) of those aged 70 years or older were examined (Fig. 1).

**Fig. 2:**
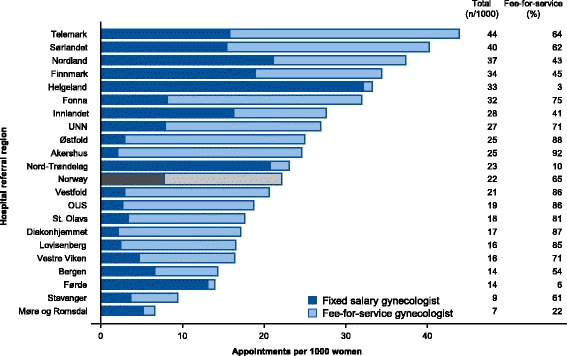
Age-standardized number of appointments for routine pelvic examination per 1000 women by hospital referral region and type of provider

**Fig. 3:**
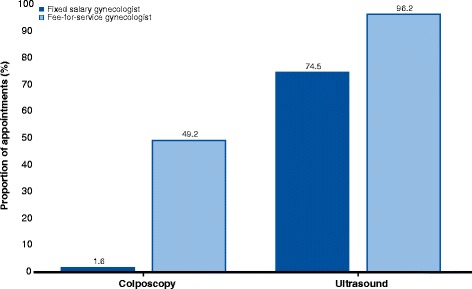
Proportion (%) of appointments with colposcopy and ultrasound in routine pelvic examinations by comparison of fixed salary and fee-for-service gynecologists

Pelvic examinations per 1000 women ranged from 6.6 to 43.9 across hospital referral regions (Fig. 2). Fee-for-service gynecologists performed two thirds (29,324) of pelvic examinations with the mean age of women examined being 1.1 years higher than at fixed salary gynecologists (47.8 vs. 46.7 years). Colposcopy was used in 1.6% (249) and ultrasound in 74.5% (11,810) of appointments at fixed salary gynecologists, while fee-for-service gynecologists used colposcopy in 49.2% (14,427) and ultrasound in 96.2% (28,216) of appointments (Fig. 3). Differences in use of colposcopy (*p* < .001) and ultrasound (*p* < .001) between provider types were statistical significant.

In addition 87.3% (29,324) of appointments in private practice had a procedural code for “complete examination performed by a specialist.”

Additional analysis showed that there were 2013 appointments within publicly funded specialized health care for cervical screening appointments annually. Cervical screening constituted 4.3% of the combined number of appointments for cervical screening and routine pelvic examinations. The use of ultrasound was equivalent in appointments for cervical screening and routine pelvic examinations (91.3 vs. 88.6%). Both fixed salary and fee-for-service gynecologists used colposcopy more frequently in appointments for cervical screening compared to routine pelvic examinations. Fixed salary gynecologists used colposcopy in 9.8% (13) of cervical screening appointments (routine pelvic examination: 1.2%). The corresponding number fee-for-service gynecologists was 69.8% (1310) (routine pelvic examination: 49.2%). Fee-for-service gynecologists performed 93.3% (1877) of cervical screening examinations. Out of these 87.4% (1641) had a reimbursement code for “complete examination performed by a specialist.” Adding cervical screening appointments to the analysis of regional variation caused minor sequence replacements for three regions while the national extent of variation did not change (Additional file 1).

In 16.5% (7472) of appointments one or more secondary diagnoses were registered. During the 3 year period there were 856 different secondary diagnoses from almost all chapters in ICD-10. The variation in use of colposcopy and ultrasound between appointments with and without secondary diagnoses was minimal compared to the differences between fixed salary and fee-for-service gynecologists (Table 2).

**Table 2:** Colposcopy and ultrasound in routine pelvic examinations by comparison of payment model and whether or not the appointment had a registered secondary diagnosis. Annual mean for the years 2014–16

	Fixed salary gynecologist		Fee-for-service gynecologist	
	Secondary diagnosis		Secondary diagnosis	
Outcomes	No (*n* = 14,314)	Yes (*n* = 1540)	No (*n* = 23,393)	Yes (*n* = 5932)
Colposcopy, % (n)	1.5% (220)	1.8% (28)	47.3% (11061)	56.8% (3366)
Ultrasound, % (n)	74.2% (10,616)	77.6% (1195)	95.7% (22,381)	98.4% (5835)

Excluding appointments with secondary diagnoses in the analysis of regional variation caused minimal sequence replacements of five regions (Additional file 2).

## Discussion

### Principal findings

Routine pelvic examinations are widespread in Norway. Annually, 22.2 per 1000 adult women received a pelvic examination that is recommended against. The variation across hospital referral regions was extensive and ranged from 6.6 to 43.9 per 1000 women. Gynecologists with fixed salaries performed colposcopy in 1.6% and ultrasound in 74.5% of appointments, while fee-for-service gynecologists performed colposcopy in 49.2% and ultrasound in 96.2% of appointments. Fee-for-service payments for gynecologists seem to drive the utilization of colposcopy and ultrasound in routine pelvic examinations.

### Interpretation

This is the first study to document the widespread use of unwarranted routine pelvic examinations in Norway. The great majority of examinations were performed on women aged 25–69 years. The Norwegian Cervical Cancer Screening Programme recommends and reminds all women between the age of 25 and 69 years to have a cytology test done every 3 years [19]. The correlation between cervix screening age and the age distribution in this study indicates that a large proportion of women with a routine pelvic examination may have had the extended examination as part of cervix screening. The number of appointments for each woman was 1.14 during the 3 years study period, which further strengthens this interpretation.

The real extent of routine pelvic examination in specialized health care seems to be higher than our study reveals, as the content of health care delivered in cervical screening appointments is equivalent to what is demonstrated in routine pelvic examinations. Pelvic examination, pelvic ultrasound and colposcopy are not indicated in asymptomatic women and are not part of The Norwegian Cervical Cancer Screening Programme, unless the result of the cytology test shows cause for concern [19]. There are separate ICD-10 codes for abnormal cervical cytological findings [15]. If women in our study actually were referred to specialized health care for routine testing within The Norwegian Cervical Cancer Screening Programme, our results demonstrate overuse of specialist health care services as cervical screening is supposed to be a primary care undertaking. This reflects a recently observed shift from primary to specialized health care for insertion of intrauterine contraception [20]. The finding of high numbers of colposcopy, ultrasound, and “complete examinations” in cervical screening appointments adds to this overuse. Based on our findings, we argue that primary care physicians should perform cervix screening.

Concomitant cervix screening cannot explain the extensive regional variation observed. Neither can differences in morbidity across the regions, as the women examined were by definition healthy. Geographical variation is shown to be associated with supply sensitive care [21]. The extent of variation in the present study points to examinations that are dependent on local health care practice and supply.

While the American College of Physicians, the Canadian Task Force on Preventive Health Care and the American Academy of Family Physicians strongly recommend against routine pelvic screening examinations, the debate is not settled. The US Preventive Services Task Force Recommendation Statement​ concludes “that the current evidence is insufficient to assess the balance of benefits and harms of performing screening pelvic examinations” [22]. The American Congress of Obstetricians and Gynecologists reaffirmed in 2016 their Committee Opinion which purpose is “to explain the need for annual assessments” albeit “at this time, this recommendation is based on expert opinion” [23].

The academic ambiguity concerning routine pelvic examinations might be reflected in our findings of extensive regional variation. As all the appointments required a referral, the regional variation might be explained by regional differences in referral pattern. However our study cannot answer if the observed variation is due to regional differences in: supply (i.e. the number of gynecologists to refer to); professional belief in and tradition for routine examinations; or the proportion of examinations performed by primary care physicians and gynecologists, respectively.

Either way, there is no pelvic screening program in Norway. Both the extensive regional variation and the extent of routine pelvic examinations per se are unwarranted in regard to the Norwegian Patients’ Rights Act [14] and in regard to the Norwegian Medical Associations concerns on opportunistic screening [24].

Health expenditures are increasing worldwide and account for more than 12% of gross domestic product in OECD countries [25]. Apart from Luxembourg, no country spends more on publicly financed health care per capita than Norway [26]. It is recognized that fee-for-service reimbursement is the most important driver of high medical expenditures in the United States [27]. Fee-for-service in primary care has been reported to be associated with more visits, diagnostic tests and referrals compared to salary payment, though evidence is limited [28]. Ransom et al. have demonstrated that elective gynecological procedures are performed more frequently under fee-for-service than capitation payment [29]. The present study supports these findings as fee-for-service gynecologists used colposcopy and ultrasound 31.2 and 1.3 times more often than gynecologists with fixed salaries, respectively. Fee-for-service gynecologists have an economic incentive to extend the examination not only through the tariff for colposcopy and ultrasound, but also through reimbursement for “complete examination.” This code was used in 87.3% of fee-for service appointments.

Theoretically, patient preferences might explain some of the differences between provider types and also the regional differences. However there is no evidence that patient preferences have much impact on regional variation [30]. It is highly unlikely that healthy women referred to fixed salary physicians opt out colposcopy while the majority of women examined by fee-for-service gynecologists actively want this procedure. Moreover colposcopy and ultrasound are advised against in the screening setting, and should not be an offer within publicly funded healthcare regardless of preferences. Our results strongly imply that fee-for-service payments for gynecologists skyrocket the use of colposcopy and drive the use of “complete examinations” and ultrasound in pelvic examinations of asymptomatic women.

Recalibrating fee-for-service payments is recommended as one measure to constrain unsustainable health care expenditures [27]. Based on our findings, we argue that reimbursements for routine pelvic examinations including complete examination, colposcopy and ultrasound in women not registered with any symptom, complain or diagnosis should be discontinued. If gynecologists perform cytology screening in healthy women, any extra reimbursement should be removed.

### Generalizability

To our knowledge, no other studies have quantified the national extent of routine pelvic examinations within publicly funded specialized health care. In Norway there has never been a national guideline recommending pelvic examination in asymptomatic women, nor a screening program for ovarian cancer. “Well-woman visits” [23] are not advocated by any Norwegian health authorities and the majority of women are unfamiliar with the practice. It is reasonable to believe that Norway scores relatively low on the number of routine pelvic examinations compared to countries with traditions and recommendation for annual assessments, and countries with a higher degree of fee-for-service-reimbursements for gynecologists.

This study only quantifies the use of pelvic examinations within publicly funded specialized health care. Private gynecologists with public funding constitute 43.5% of all private gynecologists in Norway [31]. The remainders are privately paid. The number of routine pelvic examinations paid out-of-pocket is unknown, as is the number performed by primary physicians. There is no reason to believe that privately paid gynecologists perform routine pelvic examinations any less than publicly funded gynecologists. On the contrary, privately paid gynecologists commonly advertise for routine pelvic examinations, hence, we believe that our study substantially underestimates the total amount of unwarranted pelvic examinations in Norway.

### Strengths and limitations

The major strength of the study is the inclusion of all Norwegian adult women and that the studied codes give the basis for actual reimbursements paid to hospitals and fee-for-service gynecologists. Registration and reporting of appointments is compulsory and economically important for both hospitals and fee-for-service gynecologists. Correct reporting is focused on and stressed in both settings.

There are several limitations inherent in the methodology of register studies. Code practice may vary across regions. Underreporting of secondary diagnosis is expected [32]. Fee-for-service gynecologists get reimbursement according to the procedures they perform, while fixed salary gynecologists neither get compensated personally nor get more reimbursements to the hospital by performing colposcopy or ultrasound in routine pelvic examinations. Hence, it is possible that fee-for-service gynecologists are more thorough in their reporting, and that the actual use of colposcopy and ultrasound especially in hospitals is underreported. Still, it is highly unlikely that this can explain the huge differences observed.

## Conclusions

Annually, 22.2 per 1000 adult women in Norway received a publicly funded pelvic examination that is recommended against. The variation across regions was extensive. Our results strongly indicate that fee-for-service payments for gynecologists skyrocket the use of colposcopy and increase the use of ultrasound in routine pelvic examinations. We argue that the reimbursement for these examinations should be discontinued, not only as a measure to constrain the unsustainable growth in health care expenditures, but also for the wellbeing of healthy women.

## Additional files

Additional file 1: Age-standardized number of appointments for routine pelvic examination and cervical screening per 1000 women by hospital referral region and type of provider. (PDF 146 kb)

Additional file 2: Age-standardized number of appointments for routine pelvic examination per 1000 women by hospital referral region and type of provider. Appointments with secondary diagnoses are excluded. (PDF 146 kb)

## Abbreviations

ICD-10: The International Classification of Diseases version 10
NCMP: The Norwegian Classification of Medical Procedures
NCRP: The Norwegian Classification of Radiological Procedures
NCSP: The NOMESCO Classification of Surgical Procedures
NPR: The Norwegian Patient Registry
