# Supplementary figures and images for: Extent, regional variation and impact of gynecologist payment models in routine pelvic examinations: a nationwide cross-sectional study
Source: BMC Womens Health. 2017 Nov 21;17:114. doi: 10.1186/s12905-017-0471-2 (PMC5697055; doi:10.1186/s12905-017-0471-2)

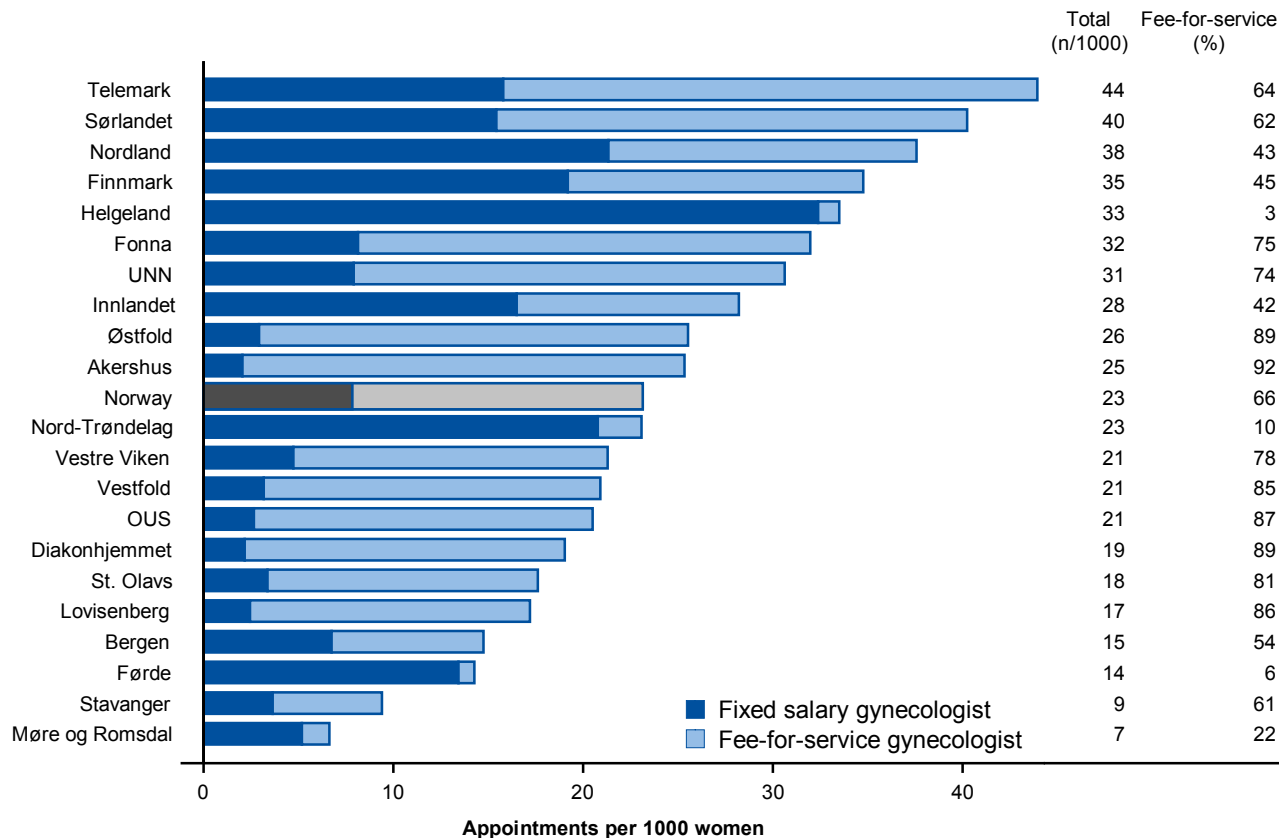

Supplement: Supplementary file 1 — Age-standardized number of appointments for routine pelvic examination and cervical screening per 1000 women by hospital referral region and type of provider. (PDF 146 kb) [file 12905_2017_471_MOESM1_ESM.pdf]

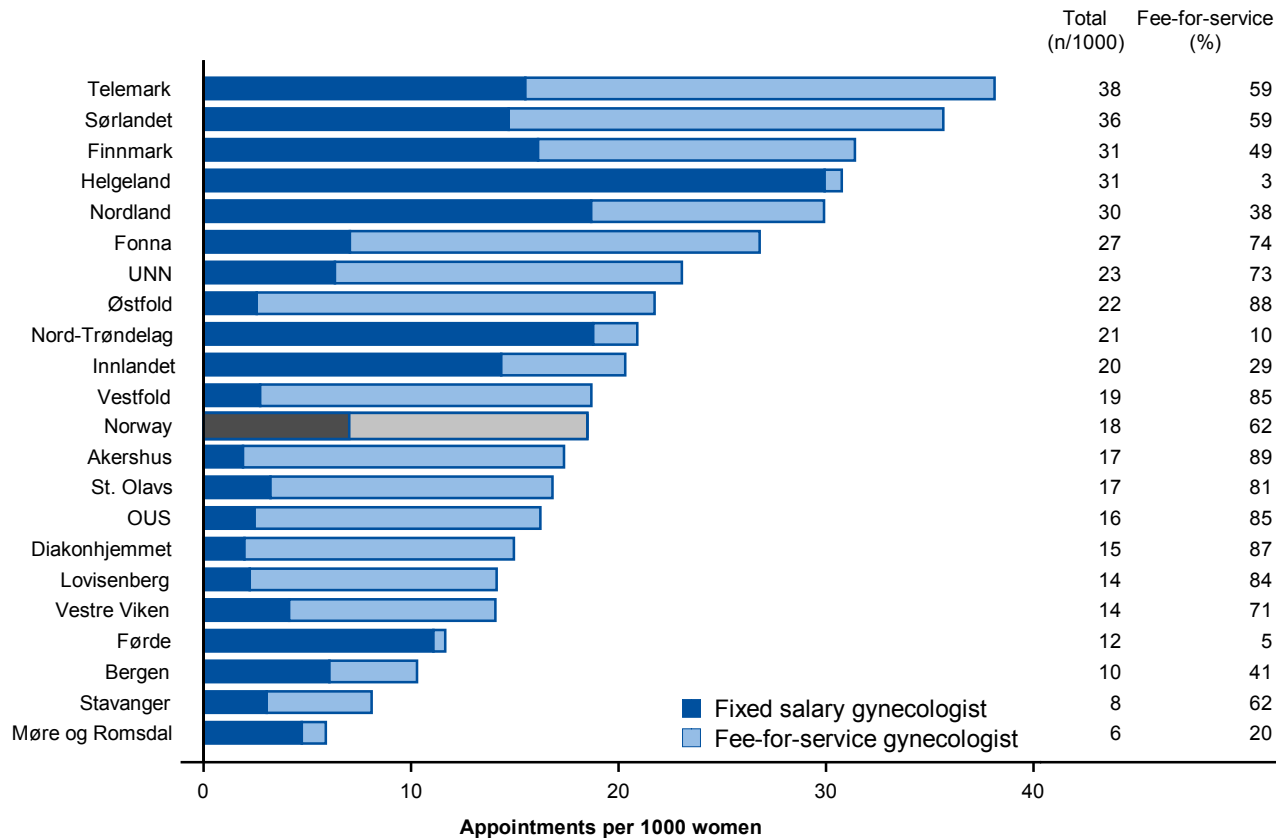

Supplement: Supplementary file 2 — Age-standardized number of appointments for routine pelvic examination per 1000 women by hospital referral region and type of provider. Appointments with secondary diagnoses are excluded. (PDF 146 kb) [file 12905_2017_471_MOESM2_ESM.pdf]
